# Analysis of bacterial contamination and the effectiveness of UV light-based reprocessing of everyday medical devices

**DOI:** 10.1371/journal.pone.0268863

**Published:** 2022-11-09

**Authors:** Stefan Alexander Rudhart, Frank Günther, Laura Isabel Dapper, Francesca Gehrt, Boris Alexander Stuck, Stephan Hoch

**Affiliations:** 1 Department of Otolaryngology, Head and Neck Surgery, University Hospital Marburg, Philipps-University, Marburg, Hessen, Germany; 2 Department of Medical Microbiology and Hygiene, University Hospital Marburg, Philipps-University, Marburg, Hessen, Germany; Annamalai University, INDIA

## Abstract

**Background:**

The reprocessing of daily used medical devices is often inadequate, making them a potential source of infection. In addition, there are usually no consistent and technically standardized procedures available for this purpose. Hence, the aim of this study is to analyze the bacterial contamination and the effectiveness of Ultraviolet light-based (UV light-based) reprocessing of daily used medical devices.

**Material and methods:**

Six different everyday medical devices (20 each; stethoscopes, tourniquets, bandage scissors, reflex hammers, tuning forks, and nystagmus glasses) were tested for bacterial contamination. All medical devices were then exposed to UV-C light for 25 seconds. Medical devices with a smooth surface were pre-cleaned with a water-based wipe. Contact samples were taken before and after reprocessing.

**Results:**

Immediately after clinical use, 104 of 120 contact samples showed an average bacterial contamination of 44.8±64.3 colony forming units (CFU) (0–300 CFU), also including potentially pathogenic bacteria. Two further culture media were completely overgrown with potentially pathogenic bacteria. The stethoscopes were found to have the highest average contamination of 90±91.6 CFU. After reprocessing, 118 of 120 samples were sterile, resulting in an average residual contamination of 0.02±0.1 CFU in two samples, whereby only bacteria of the ordinary skin flora were found.

**Conclusion:**

The present study shows the potentially clinically relevant bacterial contamination of everyday used medical devices. The reprocessing method tested here using UV light appears to be a suitable method for disinfection, especially for objects that up to now have been difficult to disinfect or cannot be disinfected in a standardized manner.

## Introduction

In the routine medical treatment, various medical devices are used daily in direct patient contact, often several of them on the same patient. Due to a lack of time resources, but above all a lack of practical reprocessing options, potentially leads to insufficient disinfection of the equipment between patient contacts [1]. Inadequately disinfected medical devices increase the risk of cross-infections and thus also of nosocomial infections, which are often difficult to treat [2]. In recent years, an increasing number of outbreaks of infectious diseases caused by multidrug-resistant pathogens have been observed in medical facilities, which have been associated, among other things, with the use of improperly reprocessed medical devices [3, 4]. Especially against the background of the current COVID 19 pandemic, where common medical devices are often used on potentially highly infectious patients, effective reprocessing of these devices becomes even more important. For some common and interdisciplinary used medical devices, such as stethoscopes, venous tourniquets or bandage scissors, microbiological examinations after usage have already been performed and in some cases hygiene recommendations have already been established [5–8]. With regard to their potential risk of infection and the requirements for their reprocessing, these everyday used medical devices are classified as non-critical medical devices according to Spaulding classification [9]. Accordingly, "low-level" reprocessing in the form of wipe disinfection after use would generally be adequate to ensure safe reusability of the medical devices [9, 10]. However, a problem in the reprocessing of these materials is usually not the effectiveness of the reprocessing procedures themselves, but their inadequate application of users, which usually results from a lack of familiarity with the disinfection method [1, 11]. In addition, due to their material properties many daily used devices are not suitable- or only suitable to a limited extent for the conventional reprocessing procedures due to their material properties. Against this background, a standardization of the reprocessing of non-critical medical devices by a user-independent and reliable reprocessing method, that can be used close to the patient and independent of the material, would be of high clinical importance.

Ultraviolet light-based (UV light-based) disinfection could be a possible solution here. The disinfecting properties of UV radiation have been known for over 120 years. Initially, it was used by the Danish physician Nils Ryberg Finsen for the treatment of infectious diseases, which was honored in 1903 with the Nobel Prize [12, 13]. After the end of the Second World War, UV lamps were mostly used for surface disinfection all over Europe. In the course of time, they were replaced by other methods, however, mainly because of their carcinogenic properties [14]. Nowadays, UV light-based reprocessing methods can be found in various areas of application, such as the disinfection of drinking water [15]. UV light offers the advantage that it has a disinfecting effect without chemical components and is therefore neither toxic nor does it influence the taste or smell of the exposed material [15]. Furthermore, bacterial resistance mechanisms or biofilm-forming properties of bacteria do not lead to a reduction of the disinfecting effect of UV light [16, 17]. The effectiveness of surface disinfection by UV-C light was already documented in previous studies by this research group on "semi-critical" instruments (rigid and flexible endoscopes) [18, 19]. In this case, a reduction of bacterial contamination by a total of log 6 could be achieved. Furthermore, almost all endoscopes were sterile and almost protein-free after reprocessing, which fulfils the regulatory requirements for "high-level" disinfection [18]. Similar results were found for flexible endoscopes without a working channel using the same technology [19]. Furthermore, the virucidal properties of the disinfection system tested here have already been investigated, which is particularly important in the current COVID 19 pandemic. In this study a significant LOG reduction was observed after only 25 seconds of irradiation [20].

The aim of the present study was therefore to determine the bacterial contamination of daily used medical devices in the context of clinical routine and the effectiveness of their UV-C light-based reprocessing.

## Material and methods

### Analyzed medical devices and reprocessing

**Fig 1:**
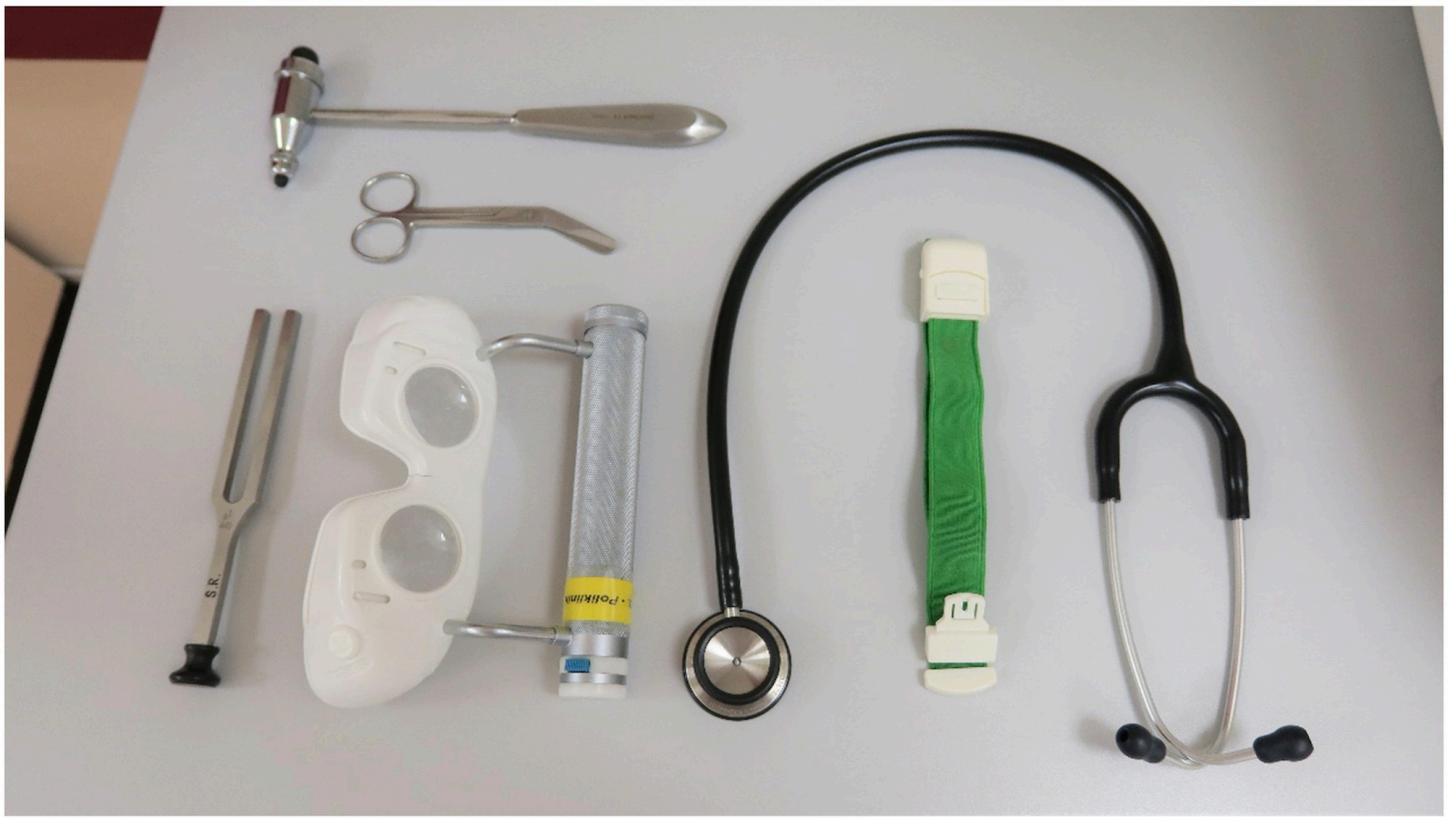
Analyzed medical products. Trömner reflex hammer, 180g (Rudolf Riester GmbH, Jungingen, Germany); bandagescissor (Lister Verbandsschere 14.5cm, Schwestern-Kaufhaus GmbH, Rheine, Germany); tuning fork a1 440 Hz with plastic base (KARL STORZ SE &Co.KG, Tuttlingen, Germany); Frenzel nystagmus glass (DEHAG Medizin-Technische Produktion GmbH & Co. KG, Rosdorf, Germany); Stethoscope (3M^™^ Littmann^®^ Classic II, 3M Deutschland GmbH, Neuss, Germany) and one-hand vein tourniquet (TIGA-MED Deutschland GmbH, Ronneburg, Germany).

For the present study, various medical devices were tested which are used daily in the context of the patient treatment in the inpatient as well as in the outpatient sector. The following medical devices were analyzed: Stethoscopes (3M^™^ Littmann^®^ Classic II, 3M Deutschland GmbH, Neuss, Germany), one-hand vein tourniquets (TIGA-MED Deutschland GmbH, Ronneburg, Germany), bandage scissors (Lister Verbandsschere 14.5cm, Schwestern-Kaufhaus GmbH, Rheine, Germany), Trömner reflex hammers, 180g (Rudolf Riester GmbH, Jungingen, Germany), Frenzel nystagmus glasses (DEHAG Medizin-Technische Produktion GmbH & Co. KG, Rosdorf, Germany) and tuning forks a1 440 Hz with plastic base (KARL STORZ SE & Co.KG, Tuttlingen, Germany) (Fig 1). Microbiological sampling of the relevant parts of the investigated items was done after previous usage on the patient by contact sampling. The analyses medical devices were taken from clinical routine, whereby each product was selected and sampled 20 times. The sampled areas of the different devices are shown in Table 1.

**Table 1:** Description of the medical devices analyzed.

Medical device	Tested area of the examined device and material	Clinical use in this study
*Stethoscope*	Stethoscope head with hard plastic diaphragm and stainless steel corpus	Auscultation of both carotid arteries
*Frenzel nystagmus glass*	Hard plastic glasses frame/edge	Clinical nystagmus tests
*Tuning fork*	Hard plastic tuning fork head	Weber Test and Rinne test
*Reflex hammer*	soft rubber and stainless steel reflex hammer head	Basic neurological reflex testing:
		Patellar (knee jerk) reflex
		Achilles (ankle jerk) reflex
		Tibialis posterior reflex
		Radius-periosteal reflex
		Triceps reflex
		Biceps reflex
*Bandage scissor*	Stainless steel cutting edge	Adjusting bandages, partially directly on the patient
Vein tourniquet	Entire vein tourniquet with latex-polyester strap and hard plastic closure	Venous congestion during blood sampling or when inserting a peripheral intravenous catheter

Medical devices used in this study, their clinical application as well as the corresponding microbiologically analyzed area and its specific manufacturing material.

With the exception of the tourniquet, all medical devices were pre-cleaned by precleaning once with a water-based wipe to remove visual contamination. For this purpose, a box of polyester dry wipes (Schülke Wipes Safe and Easy, Schülke GmbH, Norderstedt, Germany) was filled with 1.5 litres of sterile water (Ampuwa, Fresenius Kabi Germany GmbH, Bad Homburg, Germany). Due to the material properties of the venous tourniquet (absorbent material made of latex and polyester), no precleaning was done. Thereafter, the medical devices were reprocessed separately for 25 seconds using UV light (D25 UV system, UV Smart Technologies B.V., Rijswijk, Netherlands). The examined objects were positioned longitudinal to the UV lamps, according to the manufacturer’s instructions. The stethoscope and the venous tourniquets were each arranged in a circular position in the UV system for reasons of the dimensions of the UV-system. When placed in a circular arrangement, the objects were positioned in order to avoid shadowing as much as possible. Afterwards, a microbiological examination by contact sampling was done. Since an effectiveness of an UV-based reprocessing has not been proven so far, a reprocessing by an already established method (mikrozid^®^ universal wipes premium, Schülke GmbH, Norderstedt, Germany) was further used before reuse of the sampled device on the patient.

### Microbiological examination

A total of 240 microbiological samples from the tested devices were evaluated (120 before and 120 after reprocessing with UV light). For each analyzed medical device, 20 examination and reprocessing procedures were done after usage on the patient. Thus, 20 samples per analyzed medical device were evaluated each before and after reprocessing. The samples were taken by contact sampling using casein-soy peptone agar culture media (Liofilchem S.r.l., Roseto degli Abruzzi (TE), Italy), as they are common culture media for both gram-positive and gram-negative bacteria. In addition, these media were used to achieve comparable results with previous studies on UV light disinfection of endoscopes. The culture media were incubated at 37°C for 7 days. Afterwards, the bacterial contamination was measured by Matrix Assisted Laser Desorption/Ionization Time of Flight mass spectrometry (MALDI-TOF) (Bruker Daltonik, Bremen, Germany).

### The D25 UV light system

**Fig 2:**
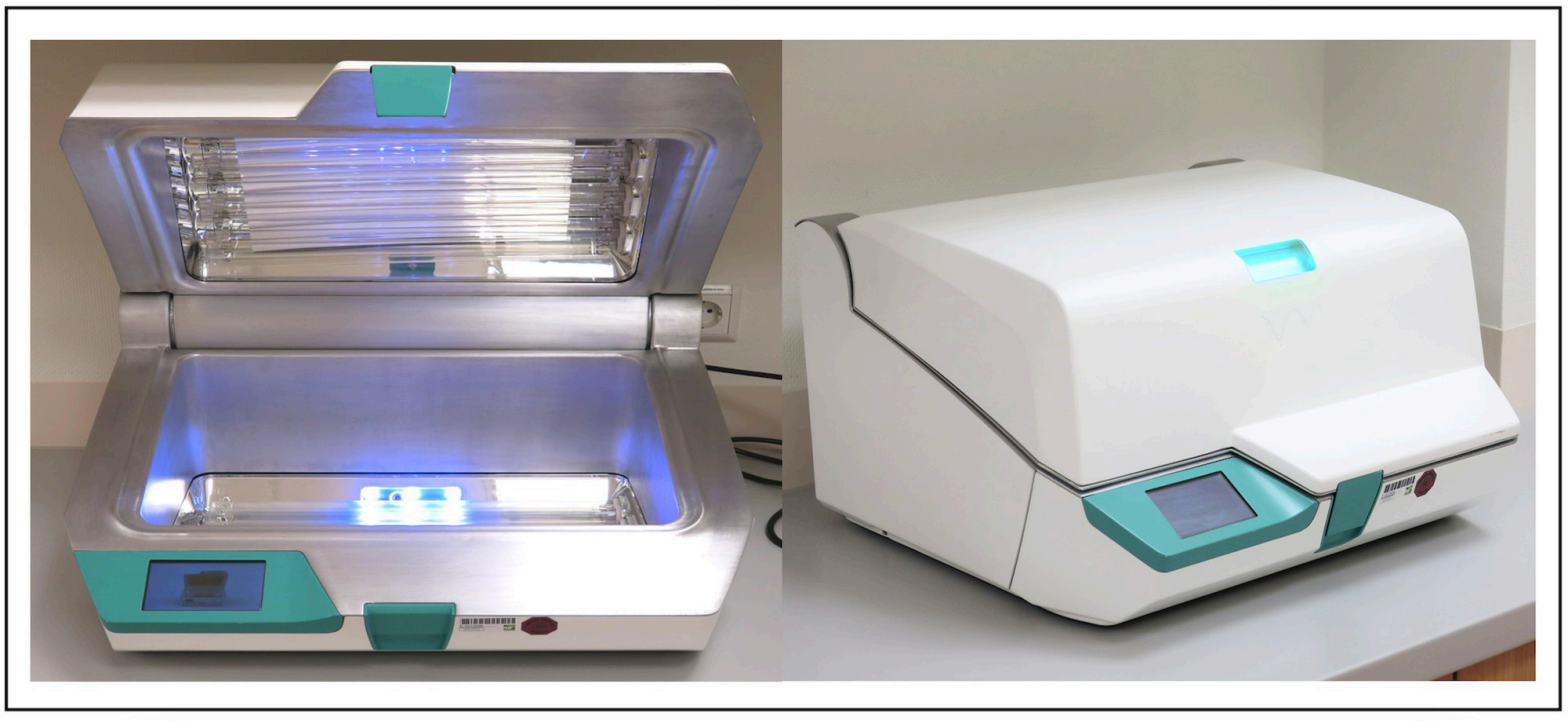
D25 UV light system. D25 UV light system in open (a) and sealed position (b).

The method of disinfection in the D25 UV light system is based on the physical properties of UV-C light with a wavelength of 253.7 nm. Each disinfection cycle over 25 seconds, a dose of 6872 μW/cm2 is applied, which, according to the manufacturer, should result in a reduction of the bacterial load by at least LOG 5. The UV-C radiation irreversibly destroys the DNA and/or RNA of the microorganisms on a molecular level when exposed to the light [21]. Besides the water-based pre-cleaning wipe, no further chemicals or liquids are needed for the treatment process. The UV system is box-shaped to prevent UV radiation from escaping and potentially harming users (Fig 2a, 2b). In addition, the UV lamps switch off directly if the system is opened during operation.

The system’s treatment chamber dimensions are 380 mm in length, 225 mm in width and 150 mm in height. If objects should exceed these dimensions, according to the manufacturer, circular positioning in the chamber is recommended, to avoid shadowing. Eight UV light-applying lamps, protected by a special glass plate, are installed on both the top and the bottom of the system. The sides of the treatment chamber are lined with reflective metal elements to ensure most efficient distribution of UV light and to prevent shadowing. For users’ safety, the disinfection chamber is sealed while UV-C light is applied. In addition, the lamps switch off immediately when the device is opened during the disinfection process.

#### Statistics and ethics vote

Descriptive statistical analysis was performed using Excel 2019 (Microsoft Corporation, Redmond, WA, USA). This study was reported to the Ethics Committee of the Department of Medicine at the Philipps University of Marburg. According to the statement of the ethics committee, no formal ethics vote was required.

## Results

**Fig 3:**
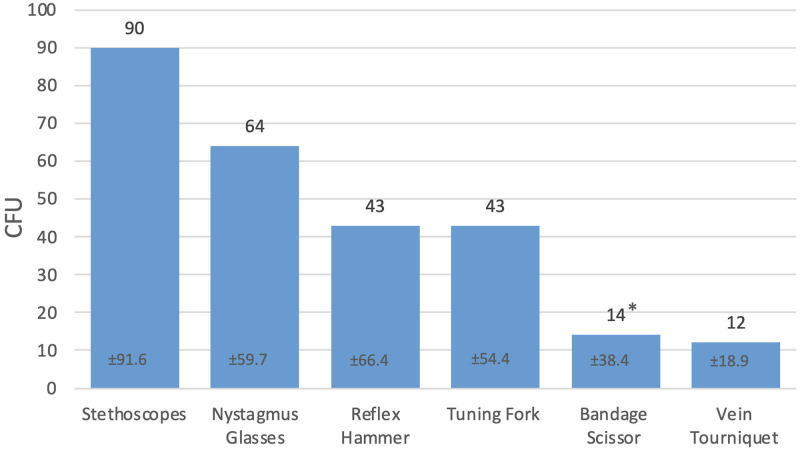
Average bacterial contamination on the analyzed medical devices without disinfection. Average bacterial contamination and standard deviation in CFU on the analyzed medical devices. * Two further culture media were completely overgrown and therefore not quantitatively evaluable.

Immediately after usage on the patient, 104 of the 120 tested medical devices were found to be bacterially contaminated. On average, the contamination was 44.8±64.3 colony forming units (CFU) (0–300 CFU) per culture medium, whereby two further culture media were completely overgrown with a bacterial lawn after incubation and were therefore no longer accessible for quantitative evaluation (Fig 3).

The stethoscopes showed the highest average contamination with 90±91.6 CFU (0–300 CFU), followed by the nystagmus glasses with an average of 64±59.7 CFU (0–180 CFU). The tuning forks and reflex hammers each had an average bacterial contamination of 43±54.4 and ±66.4 CFU (0–150; 0–300 CFU), respectively. 18 of the 20 bandage scissors showed an average contamination of 14±38.4 CFU (0–160 CFU), whereas two further culture media were completely overgrown with a bacterial lawn and therefore could not be evaluated quantitatively. Both culture media were overgrown with *Pseudomonas aeruginosa* and *Staphylococcus species*, while *Enterococcus faecium* was also found on one of the two culture media. The lowest average contamination was found on the vein tourniquets with an average of 12±18.9 CFU (0–80 CFU). It can be noted that after usage on the patient, in addition to bacteria of the ordinary skin flora, also potentially pathogenic germs such as *Pseudomonas aeruginosa*, *Enterococcus faecium* and *Corynebacterium species* were found on the reflex hammers, bandage scissors and vein tourniquets. A detailed evaluation of the bacterial contamination is shown in Tables 2 and 3.

**Table 2:** Identified bacteria before and after UV disinfection.

Medical device	Before Reprocessing		After UV-disinfection	
	Identified bacteria	Number of samples (n)	Identified bacteria	Number of samples (n)
Stethoscope	*Staphylococcus species*	17		
	*Staphylococcus haemoliticus*	4		
	*Staphylococcus aureus*	1		
	*Bacillus species*	1		
Frenzel nystagmus glass	*Staphylococcus species*	18		
	*Staphylococcus aureus*	3		
Tuning fork	*Staphylococcus species*	18		
	*Paenibacillus species*	3		
	*Staphylococcus haemoliticus*	1		
	*Bacillus species*	1		
Reflex hammer	*Staphylococcus species*	18		
	*Enterococcus faecium*	4		
	*Corynebacterium species*	2		
	*Roseomonas mucosa*	1		
Bandage scissor	*Staphylococcus species*	13	*Staphylococcus species*	1
	*Pseudomonas aeruginosa*	3		
	*Enterococcus faecium*	2		
	*Bacillus species*	1		
Vein tourniquet	*Staphylococcus species*	13	*Bacillus species*	1
	*Bacillus species*	4		
	*Enterococcus faecium*	1		
	*Micrococcus luteus*	1		
	*Pantoea species*	1		

Identified bacteria depending on the detection frequency on the examined medical devices before and after UV disinfection.

**Table 3:** Frequency distribution of the bacteria identified.

Identified bacterium	Absolute number (%)
*Staphylococcus species*	106 (80.3)
*Enterococcus faecium*	7 (5.3)
*Bacillus species*	7 (5.3)
*Pseudomonas aeruginosa*	3 (2.3)
*Corynebacterium species*	3 (2.3)
*Paenibacillus species*	3 (2.3)
*Micrococcus luteus*	1 (0.8)
*Pantoea species*	1 (0.8)
*Roseomonas mucosa*	1 (0.8)

Frequency distribution of the bacteria identified on the tested medical devices without UV disinfection.

After reprocessing with UV-C light, 118 of 120 tested medical devices were even sterile. The average contamination on the tested objects was 0.02 CFU (± 0.1; 0–1 CFU). After disinfection, a residual contamination of 1 CFU each was found on one bandage scissor and one vein tourniquet. *Bacillus species* were detected on the surface of the tourniquet and *Staphylococcus species* on the surface of the bandage scissors (Table 2). However, both bacteria can be classified as part of the ordinary skin flora.

## Discussion

Cross-infections and associated outbreaks of potentially highly pathogenic bacteria in the context of non-critical medical devices have been described several times in the literature [22–24]. So far, the containment of those cross-infections caused by medical devices has not been successful, even in western countries as e.g. Germany [22]. It is assumed that cross-infections are often caused by an insufficient disinfection of the medical devices between different patients [5].

Thus, in literature, 12–47% of the employees from specialized hospitals declared that they never clean their stethoscope or only clean it once a year [25]. In surveys among medical students at a university hospital in Slovakia, as many as 94% of respondents stated that they had never disinfected their stethoscope since purchasing it [1]. As described by other authors, this results in contamination rates of up to 75% of the stethoscopes immediately before reuse on patients [26]. In addition to education campaigns, simple and fast disinfection procedures, that allow on site disinfection could improve this situation. According to Spauldings, disinfection with wipes are sufficient for disinfecting non-critical medical devices, therefore, disinfection wipes are most often used for disinfection of the medical devices examined here [9]. However, at this point it should be noted that the results of disinfection wipes are always user-dependent due to manual handling. In the literature, a lack of familiarity and thus incorrect manual handling of the reprocessing methods has been identified as the main reason for insufficient disinfection of medical devices [1]. Another problem in the manual disinfection of medical devices is the infection risk and the risk of allergic reactions from chemicals for the users. Moreover, conventional automated reprocessing methods are not suitable for all of the above-mentioned medical devices due to their material properties. A standardized, non-user-dependent UV-C radiation-based disinfection method could be a safe and effective alternative here. In general, it should be mentioned that UV light radiation is a potential source of danger for the user due to the physical damage of DNA, e.g. RNA, which, amongst others may result in a carcinogenic effect [14]. However, the system described here is box-based designed and sealed while applying UV light radiation, which prevents radiation from escaping. As a further safety measure, the lamps switch off as soon as the system is opened during the disinfection process.

In the present study, significant bacterial contamination was found on all medical devices analyzed, in some cases also including potential pathogenic germs. On the surface of the stethoscopes the highest average bacterial contamination was found. The results of this study are consistent with the available data from international literature. Here, stethoscopes in larger western hospitals showed an average bacterial contamination of 27 to 158 CFU, depending on the publication, with an average of 85.1% of stethoscopes showing a relevant bacterial contamination on their surface [27–32]. Accordingly, the contamination level of 90 CFU on average found in this study seems to be in line with the literature. Similar contamination rates as found in the present study are also described in the literature for tourniquets and bandage scissors in hospitals in western countries [33, 34]. For the other medical devices examined here, no information on the frequency and extent of bacterial contamination after usage is available in the literature.

The bacteria identified in the present study are mainly attributed to the normal skin flora [35]. However, potentially pathogenic bacteria such as *Enterocuccus faecium* or *Pseudomonas aeruginosa*, which may cause relevant infections, were also detected on medical devices in this study before disinfection. A resistance test of the bacteria was not performed in the present study due to the proven effectiveness of UV radiation against bacterial resistance mechanisms [16].

After disinfection with the D25, only two of the medical devices examined showed minimal residual contamination, whereby only bacteria of the skin flora were found. In earlier studies of our department, the D25 UV-C light system examined here had already shown promising results in reprocessing of rigid endoscopes without working channel [18]. An average contamination of 66,908 CFU was found on the endoscopes before disinfection. Furthermore, similar to the present study also potentially pathogenic germs such as *Pseudomonas aeruginosa* as well as germs of the normal skin e.g. mucosal flora were found. After UV-based disinfection, an average of only 0.12 CFU was detectable on the endoscopes analyzed, whereby only bacteria of the normal mucosal flora were found. With correspondingly positioned test bodies, the light system achieved an absolute germ reduction of 6 log levels and was thus able to fulfil the requirements of the legislator for the reprocessing of semi-critical medical devices, tested so far. In another UV light-based disinfection system of the Dutch company UV Smart, similar results were achieved in the reprocessing of flexible ENT endoscopes without working channel [19]. After use on a patient, the flexible endoscopes showed an average contamination of 916.7 CFU with potentially pathogenic bacteria. After disinfection with the UV-C system, an average of 0.28 CFU was found on the endoscopes, while the remaining endoscopes showed 0 CFU. The bacteria found after reprocessing can all be attributed to the mucosal flora. However, the tests were performed under clinical conditions. Thus, the residual contamination might be explained by artificial contamination due to handling of the endoscope after disinfection. In addition, all test specimens showed no further contamination after UV exposure, resulting in an absolute bacterial count reduction of 10^7^. It is noticeable that bacteria of the normal skin flora or mucosal flora, which could also be exposed to natural UV light, can be detected after disinfection in our studies with high power UV light. Accordingly, it might be possible that these bacteria are already less sensitive to UV radiation compared to bacteria, e.g. of the intestinal tract.

No scientific publications are currently available on viral contamination or a virucidal effect of UV-based disinfection on the medical products used in the present study. However, it remains unclear whether viral contamination is relevant in the context of the present study or the usual use of the medical devices examined. However, a publication by our own working group with rigid endoscopes showed a significant reduction of MS-2 bacteriophages as a surrogate marker of virological contamination by 3.0 log levels after 25 seconds, 4.2 log levels after 50 seconds of irradiation and by 5.9 log levels after 75 seconds of UV irradiation with the D25. This means, that even after 25 seconds more than 99% of the bacteriophages were eliminated [20].

According to the literature, besides viral contamination, fungal contamination is difficult to address by UV light [36]. In a previous study, up to 5 times longer irradiation was necessary for 99.9% denaturation of fungi compared to bacteria. Nevertheless, a 99.9% disinfection performance was achieved for fungi after only 15–30 seconds [37]. It should be mentioned that the UV light source used in this trial was approx. 2 times stronger than the lamp used in our study. However, fungi are generally sensitive to UV-radiation as their denaturation is mainly influenced by the power of the UV light source and time of irradiation. Since in the present study the bactericidal properties of UV C radiation were analyzed, the fungicidal effect was not tested. Further studies on the fungicidal efficacy of the UV light system used are already projected.

The effectiveness of UV-C radiation against multi-resistant and biofilm-forming bacteria is an advantage of the disinfection method tested here compared to other reprocessing methods e.g. disinfection by wipes [16, 17]. Compared to some chemical disinfection methods, however, there is the disadvantage that UV radiation cannot penetrate solid substances or turbid liquids. Therefore, the manufacturer recommends a water-based pre-cleaning by washing once with a water-soaked wipe for objects with a hard surface before disinfection. It must be mentioned at this point that certain areas of the medical devices with undercuts (e.g. the joint of bandage scissors) might probably show a punctually higher contamination after reprocessing due to shadowing. However, a microbiological examination of this area was only possible to a limited extent due to the poor accessibility caused by the devices design. Whether this is clinically relevant remains unclear, as these parts of the medical device are not in direct contact with the patient. With regard to time aspects of the reprocessing methods, the D25 UV system and wipe disinfection methods do not differ relevantly due to the exposure time of their chemical components in case of the wipes, each with a total duration of approx. 1 minute, considering a 25-second precleaning for UV-based disinfection. In the present study, a water-based pre-cleaning was used for all items, with the exception of the venous tourniquets, due to its surface structure. It should be noted, that this did not have a negative effect on the results of the disinfection. Because to our knowledge, it is not generally clear yet which amount of germ reduction can be achieved by pre-cleaning [38, 39]. However, the results presented here suggest a negligible precleaning effect of the water-based wipe used, as long as there is no gross contamination.

## Conclusion

Even medical devices classified as "non-critical" carry a potential risk of cross-infection after use on the patient. Standardized UV-C light-based reprocessing significantly reduces the bacterial contamination and largely eliminates user-dependent influences during disinfection. Disinfection by UV-C light is thus an effective reprocessing method for clinical routine.

## Supporting information

S1 Data (XLSX)
